# Wafer‐Scale Electroactive Nanoporous Silicon: Large and Fully Reversible Electrochemo‐Mechanical Actuation in Aqueous Electrolytes

**DOI:** 10.1002/adma.202105923

**Published:** 2021-10-22

**Authors:** Manuel Brinker, Patrick Huber

**Affiliations:** ^1^ Institute for Materials and X‐Ray Physics Hamburg University of Technology 21073 Hamburg Germany; ^2^ Center for X‐Ray and Nano Science CXNS Deutsches Elektronen‐Synchrotron DESY 22607 Hamburg Germany; ^3^ Center for Hybrid Nanostructures CHyN University of Hamburg 22607 Hamburg Germany

**Keywords:** actuorics, cyclic voltammetry, electrochemical characterization, laser cantilever bending, nanoporous media

## Abstract

Nanoporosity in silicon results in interface‐dominated mechanics, fluidics, and photonics that are often superior to the ones of the bulk material. However, their active control, for example, by electronic stimuli, is challenging due to the absence of intrinsic piezoelectricity in the base material. Here, for large‐scale nanoporous silicon cantilevers wetted by aqueous electrolytes, electrosorption‐induced mechanical stress generation of up to 600 kPa that is reversible and adjustable at will by potential variations of ≈1 V is shown. Laser cantilever bending experiments in combination with in operando voltammetry and step coulombmetry allow this large electro‐actuation to be traced to the concerted action of 100 billions of parallel nanopores per square centimeter cross‐section and determination of the capacitive charge–stress coupling parameter upon ion adsorption and desorption as well as the intimately related stress actuation dynamics for perchloric and isotonic saline solutions. A comparison with planar silicon surfaces reveals mechanistic insights on the observed electrocapillarity (Hellmann–Feynman interactions) with respect to the importance of oxide formation and wall roughness on the single‐nanopore scale. The observation of robust electrochemo‐mechanical actuation in a mainstream semiconductor with wafer‐scale, self‐organized nanoporosity opens up novel opportunities for on‐chip integrated stress generation and actuorics at exceptionally low operation voltages.

## Graphical abstract

For large‐scale nanoporous silicon cantilevers in aqueous electrolytes, with laser cantilever bending experiments, electrosorption‐induced mechanical stress generation of up to 600 kPa that is reversible and adjustable at will by low operation voltages of 1 V is demonstrated. A comparison with planar silicon surfaces reveals mechanistic insights on the observed electrocapillarity upon ion adsorption and desorption.

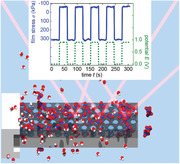

## 1 Introduction

Porous silicon has been attracting much attention since the discovery of self‐organized porosity in a monolithic semiconductor.^[^ ^1^, ^2^, ^3^, ^4^, ^5^, ^6^, ^7^ ^]^ It provides a single‐crystalline medium with anisotropic pores for the fundamental study of nanoconfinement on the structure and dynamics of matter.^[^ ^7^, ^8^, ^9^, ^10^, ^11^, ^12^ ^]^ Interface‐dominated optical, electrical, and thermal properties attract the attention of the applied sciences in fields ranging from in vivo^[^ ^4^ ^]^ and opto‐electronics^[^ ^13^ ^]^ via biosensing^[^ ^6^, ^14^ ^]^ to energy storage^[^ ^15^, ^16^ ^]^ and harvesting.^[^ ^17^ ^]^ Silicon is one of the most abundant elements in the earths crust and is available in outstanding qualities. An integration of wafer‐scale porous silicon into electrical circuit designs already existing can reasonably be achieved, as semiconductor devices are predominantly fabricated out of bulk silicon.^[^ ^6^ ^]^ Furthermore, porous silicon has been found to be biocompatible^[^ ^18^ ^]^ and a lot of functionalization schemes exist, which are using subsequent treatments to change, extend, or enhance its properties by incorporating additional functional materials^[^ ^4^, ^19^, ^20^, ^21^, ^22^, ^23^, ^24^ ^]^ or, as recently shown, by laser writing directly in pore space.^[^ ^25^ ^]^ In particular, functionalization with liquids offers a plethora of opportunities to tune properties and to create adaptive hybrids, where the soft, dynamic filling, affected by confinement, provides novel functionalities, but the rigid, semiconducting scaffold structure provides mechanical robustness on the macroscopic scale.^[^ ^24^, ^26^ ^]^ An aspect that has not yet prompted significant research is the mechanics and particularly a functionalization of porous silicon as an actuator material. Porous silicon among other, different porous media has been investigated with a focus on humidity and gas sorption‐induced actuation.^[^ ^27^, ^28^, ^29^ ^]^ Also liquid adsorption‐induced deformation of mesoporous silicon has been explored^[^ ^9^, ^30^, ^31^, ^32^ ^]^ to study the mechanical properties of mesoporous silicon or its functionalization with artificial muscle molecules.^[^ ^23^ ^]^ Another promising direction of research could be actuation mechanisms due to an electrochemical process within the material or on its surface.^[^ ^33^, ^34^, ^35^ ^]^ Given the absence of intrinsic piezoelectricity in silicon, an actuator functionality of silicon has been so far achieved either by piezo‐ceramic thin‐film on‐silicon coatings or epitaxial heterostructures, most prominently in the context of nano‐ and micromechanical systems (NEMS/MEMS).^[^ ^36^ ^]^

We follow here the strategy to integrate mechanical actuation into porous silicon by exploiting electrical potential‐induced surface stress at the internal pore surfaces, as it has been extensively studied in the domain of porous metal actuation.^[^ ^37^, ^38^ ^]^ The interface between a conductor immersed in an electrolyte solution, that is, an ionic conductor, can be electrically polarized by the formation of a Helmholtz double layer.^[^ ^39^ ^]^ Thus, the accumulated charge (*q*—surface charge density per area) on the electrode surface leads to a change in surface stress *f*. So,

(1) δf=ςδq

where ς = d*f*/d*q*|_*e*_ denotes the electrocapillarity coupling parameter and *e* the tangential strain per area. Essentially, the accumulation of charge carriers influences the bonds at the surface of the solid in the in‐plane direction and normal to it and a charge reorganization ensues which leads to electrostatic Hellmann–Feynman forces on the surface ions.^[^ ^40^ ^]^ Ab initio calculations confirm that such an electrochemo‐mechanical coupling effect can generally also be observed at silicon surfaces.^[^ ^41^ ^]^ The change in surface stress in itself can induce an actuation effect of a nonporous, bulk material, as already demonstrated for a clean gold surface.^[^ ^42^ ^]^ Platinum is another metal that has shown a similar electrochemical actuation due to an evolving surface tension by the accumulation of charge carriers.^[^ ^43^ ^]^ In this study, the fundamental relation of surface stress and accumulated charge and the resulting potential driven actuation is demonstrated for the first time for porous and planar, nonporous silicon surfaces.

For bulk materials this actuation effect is comparatively small, due to their small surface to volume ratio. For nanoporous solids with a significant internal surface area though, a change in surface stress can induce a considerable strain of the entire porous solid.^[^ ^44^ ^]^ In thermodynamics, a Maxwell relation then equates the charge‐response of *f* to the potential response on tangential strain *e* ^[^ ^45^, ^46^ ^]^

(2) $$\varsigma ={\frac{df}{dq}|}_{e}={\frac{dE}{de}|}_{q}$$

The microscopic description of these phenomena can be employed on macroscopic porous materials, by considering it as an effective medium and normalizing to volume *V*‐designated quantities. Thus, an effective strain–charge actuation coefficient is introduced, as *A* ^*^ = dε/d*q*_V_|_*T*_, where *T* is the load per cross‐sectional area. By means of *A* ^*^ the performance of the porous silicon's electrochemical actuation is assessed.

Therefore, the porous silicon electrode has to comply with the precondition that it exhibits polarizability. It means that a finite potential range in an electrochemical measurement exists where electrolyte charge carriers exclusively accumulate on the electrode surface in an electric double layer.^[^ ^47^ ^]^ In contrast, Faradaic reactions and thus currents occur in an electrochemical reaction of the involved species.^[^ ^45^ ^]^ The resulting effect of the double layer on the surface stress may vary. Metals exhibit a high conductivity and therefore charge carriers of the opposite sign accumulate at the metals surface in an Å‐thin layer to screen the material from the electrical field induced by the double layer.^[^ ^44^ ^]^ By contrast, the screening layer within a semiconductor can grow to several 100 Å.^[^ ^48^ ^]^ Moreover, the respective porous structure of nanoporous materials may differ greatly. The noble‐metal‐based nanoporous gold has an entirely different porous structure than porous silicon investigated in this study. The structure of nanoporous gold is formed by so‐called ligaments, which are interconnected in an isotropically oriented network. The porous silicon investigated here on the other hand has a highly anisotropic porous structure, which consists of parallel pores, orthogonally oriented to the silicon surface.

Overall, here we combine surface stress‐induced electrochemical actuation in planar bulk and porous silicon and the assessment of their respective properties.

## 2 Results

### 2.1 Electrochemo‐Mechanical Actuation of Nanoporous Silicon

**Figure 1:**
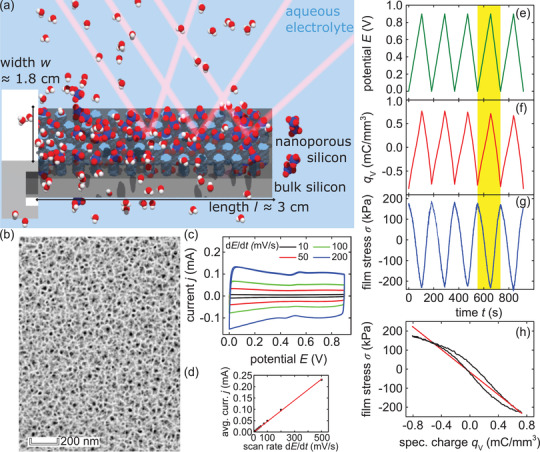
In operando laser cantilever bending experiments on electrosorption‐induced actuation of nanoporous silicon immersed in aqueous electrolyte. a) Schematic illustration of the in situ cantilever bending measurement on a porous silicon sample (light blue pores) with a bulk silicon layer underneath (light gray) in an aqueous (red, white molecules) perchloric acid (blue, red molecules) electrolyte solution. The length and width of the surface area in contact with the electrolyte solution are *l* = 2.990 cm and a width of *w* = 1.796 cm. b) TEM image of p‐doped porous silicon. c) Graph depicting exemplary cyclic voltammetry measurements of a porous silicon sample in $1\;\text{mol}\;L^{-1}\;{\text{HClO}}_{4}$ electrolyte solution. The current *j* is plotted against the applied potential *E* in the range of 0.0– 0.9 V, measured versus the standard hydrogen electrode (SHE). The potential scan rate is increased from 10 to 200 mV s^−1^. d) Graph depicting values for the current *j* from the cyclic voltammetry measurements depicted in (c), averaged in the potential range of 0.35–0.45 V. The values are plotted against increasing potential scan rates d*E*/d*t* from 10 to 500 mV s^−1^. The red linear regression line yields the capacitance *c* = 0.466 ± 0.004 mF as the slope. On the right side, the respective curves of the electrochemical actuation measurement with a scan rate of 10 mV s^−1^, are shown. e) 5 representative potential cycles *E*, f) the resulting volume specific charge *q*_V_, and g) the introduced change in film stress σ in the porous silicon layer. h) Change in film stress σ versus deposited volume specific charge *q*_V_ averaged over the 5 cycles and the linear fit to determine the stress–charge coupling parameter ξ.

The first part of the study will deal with the electrochemical and actuation properties of porous silicon. A transmission electron microscopy (TEM) image of a cross‐section of the resulting p‐doped porous silicon is depicted in **Figure** **1**b.

The fabrication process is described in detail in the Experimental Section. The TEM cross‐section shows that the pore network resembles a randomized honeycomb structure, as discussed in more detail in ref. ^[^ ^23^ ^]^. A scanning electron microscopy (SEM) image of the sample material is shown in Figure S1, Supporting Information. Another SEM image of the entire porous sample profile gives the thickness of the porous silicon layer as *h*_f_ = 630 nm. The total thickness of the sample amounts to 110 ± 2 μm. Thus, the remaining bulk silicon layer has a thickness of 109.37 μm. The volume of the porous silicon layer is then $V_{\text{pSi}}=l\times w\times h_{f}=0.338\;{\text{mm}}^{3}$, where *l* and *w* denote the length and width of the sample, further defined in the Experimental Section. A nitrogen sorption isotherme, shown in Figure S2, Supporting Information, results in a mean pore radius of *r* = 3.36 nm, an internal surface area *A*_por_ = 4.003 m^2^, and a porous volume of *V*_por_ = 0.009 cm^3^, which corresponds to a porosity of 54%.

The porous silicon sample is installed in the cantilever bending setup and immersed in 1 m HClO_4_ electrolyte solution, as sketched in Figure 1a. An initial electrochemical characterization by cyclic voltammetry (CV) measurements is conducted to examine if porous silicon can be used as a polarizable electrode. The respective measurement is shown in Figure S3, Supporting Information. It becomes apparent that the as‐fabricated porous silicon is subjected to an oxidation process. Thereby, applying a potential leads to a gradually decreasing anodic oxidation of the porous silicon walls.^[^ ^48^, ^49^, ^50^ ^]^ The electrochemically irreversible character of the oxidation is further discussed in the Supporting Information. The oxidative currents have to be drawn to a close to foreground the capacitive characteristics of the material. Therefore, a constant potential of 1.2 V is applied for 20 h. Following this preparation step, a slightly constrained potential range of 0–0.9 V will be explored in the following with the accompanying cantilever bending measurements. Note that this controlled silicon oxidation is solely performed to establish electrochemically robust surface conditions. It is not related to the reversible electrosorption‐induced actuation experiments discussed below. In particular, it is irreversible under the thereby employed electrochemical conditions. Moreover, we did not apply higher voltages, as the resulting formation of thick oxide layers (beyond 10 nm)^[^ ^48^, ^51^ ^]^ would have resulted in a complete oxidation of the pore walls and thus in a nonconductive porous layer. Hence, a capacitive charging of the nanopores would have been impossible.

Figure 1c shows cyclic voltammetry measurements in the specific potential range of 0.0–0.9 V with different scan rates from 10 to 200 mV s^−1^ for the oxidized porous silicon sample. Increasing the potential *E* from the lower vertex, causes the current *j* to increase instantly to a near constant value. Vice versa, the current quickly decreases to its negative counterpart, when the sweep direction is changed at the upper vertex. Thus, the course resembles a square around zero. This behavior holds true for the depicted courses for all scan rates d*E*/d*t*, albeit larger scan rates from 100 mV s^−1^ on exhibit a slight decrease from the lower vertex of the CV. In particular, neither an increasingly extended time until constant current, nor a linearly increasing current, instead of a constant current, is apparent. By this means, the sample seemingly shows no sign of a diffusion limited kinetic of the charge carrier up to 200 mV s^−1^.^[^ ^52^ ^]^ Thus, the charge carriers move to the working electrode, that is, the sample, in a constant flow to counterbalance an increasing potential. The actual potential is not affecting the amplitude of the current though, just the sweep direction and scan rate. The capacitance *c* of the sample determines the constant current value. The rate of the potential change d*E*/d*t* adjusts the current according to the capacitance *c* = d*j*d*t*/d*E*.^[^ ^53^ ^]^ Hence, it is possible to extract the sample's capacitance *c* by a linear regression of *j* versus an increasing scan rate. The absolute value of the current for each CV for both sweep directions is averaged in the range of 0.35–0.45 V and plotted versus the respective scan rate. The resulting plot is depicted in Figure 1d. A linear dependence is visible up to scan rates of 500 mV s^−1^. The capacitance is then obtained by a linear fit to the data and amounts to *c* = 0.466 ± 0.004 mF. The overall electrochemical analysis is evidence of the stable, capacitive features of the oxidized porous silicon electrode and thus the material can be considered as polarizable. For a complete electrochemical characterization the determination of the potential of zero charge would be ideal, albeit challenging.^[^ ^42^, ^54^, ^55^ ^]^ Recording the change in film stress σ during a CV measurement allows a detailed characterization of the electrochemical actuation of the sample. The respective measurements are depicted in Figure 1e–h. While the applied potential is reversibly changed from 0.0 to 0.9 V with a scan rate of 10 mV s^−1^ (see Figure 1e), the current is measured and hence the transferred charge can be quantified. It is normalized to the sample volume *V*_pSi_ and henceforth referred to as the volume specific charge *q*_V_, depicted in Figure 1f. The course of *q*_V_ clearly linearly coincides with the applied potential *E*. Thus, increasing the potential leads to an increased accumulation of charge carriers in the electrochemical double layer and vice versa, a decreasing potential expels charge carriers. Similarly, the change in film stress σ linearly follows the potential and the specific charge but with a negative proportionality, see Figure 1g. So, an increasing potential leads to an accumulation of charge carriers and is then responsible for a decrease in film stress σ, which is caused by a contraction of the porous silicon layer. Subsequently, a decreasing potential deprives the double layer of charge carriers and results in an expansion of the sample and thus an increase in film stress σ. The amplitude of σ exhibits no sign of a decrease and reversibly moves from a level of −210 to 170 kPa from cycle to cycle. Thus, our experiments demonstrate a robust and reversible electrosorption‐induced actuation in porous silicon.

Additionally, by averaging σ and *q*_V_ over the 5 cycles, further details can be inferred about the electrochemical actuation. The average yields a peak‐to‐peak amplitude of the film stress σ_avg_ = 406.27 ± 0.3 kPa and of the charge $q_{V_{\text{avg}}}=1.54\pm 0.04\;\text{mC}\;{\text{mm}}^{-3}$. Figure 1h shows the average film stress σ plotted against the accumulated specific charge *q*_V_ in the associated potential range of 0.0–0.9 V. It is visible, that the relation between the two is not entirely linear, but has a slightly smaller slope in the beginning from ≈−0.8 to −0.4 mC mm^−3^. Moreover, it shows a hysteresis from −0.4 mC mm^−3^ on. This behavior is potentially the result of retardation in the stress response on the charge accumulation. Nonetheless, a linear fit to the data yields the corresponding stress–charge coupling parameter ξ = −296 ± 1 mV. ξ is a key materials parameter to asses the electrochemo‐mechanical coupling that drives the actuation of porous silicon.

**Figure 2:**
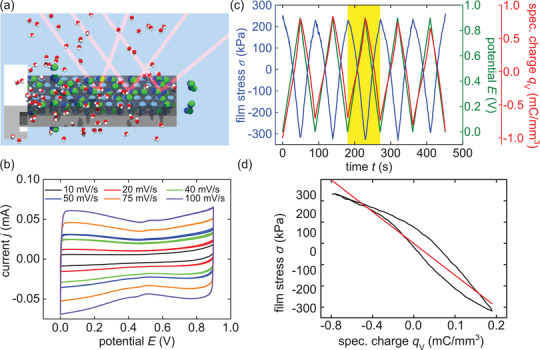
In operando laser cantilever bending experiments on electrosorption‐induced actuation of nanoporous silicon immersed in isotonic saline solution. a) Schematic illustration of the cantilever bending measurement on a porous silicon sample in aqueous (red, white molecules) NaCl (green, blue molecules) electrolyte solution. b) CV measurements in an isotonic saline solution (154 mmol L^−1^ NaCl aq.) with different scan rates in the potential region of 0.0–0.9 V. The ascertained capacitance is *c* = 0.473 ± 0.003 mF. c) Depicted are the respective curves of the electrochemical actuation measurement with a scan rate of 10 mV s^−1^ in the same NaCl aqueous electrolyte solution versus time, as five representative potential cycles of *E* (green), *q*_V_ (red), and σ (blue). The film stress σ averaged over 5 cycles versus deposited volume specific charge *q*_V_ yields ξ = −374 ± 2 mV via the respective linear fit. d) Averaged film stress σ versus deposited volume specific charge *q*_V_ and the linear fit to determine the stress–charge coupling parameter ξ = −374 ± 2 mV.

To explore the potential biomedical application of this process, analogous measurements are repeated with an aqueous electrolyte solution of isotonic sodium chloride, as omnipresent in (bio‐)medical environments, see **Figure** **2**a. Figure 2b shows the respective CVs with scan rates from 10 to 100 mV s^−1^. The CV measurement are conducted in the same potential range, exhibiting the characteristics of a capacitive regime as well. Through the fit of the averaged absolute current of both sweep directions between 0.35 and 0.45 V versus the scan rate, the capacitance of the sample is determined. It yields a value of *c* = 0.473 ± 0.003 mF, which is in excellent agreement with the value obtained with HClO_4_ as the electrolyte. Figure S4, Supporting Information shows the fit for *c*.

In Figure 2c, the electrochemical actuation measurement in the corresponding potential range with the NaCl electrolyte solution and a scan rate of 10 mV s^−1^ is displayed. The film stress σ shows the same characteristics as with HClO_4_. It follows the potential and the accumulated specific charge *q*_V_ linearly with a negative sign. Averaging the stress over the cycles results in a σ of 503.2 ± 0.5 kPa, while the accumulated volume specific charge *q*_V_ remains in the same range of −0.8 to +0.8 mm^3^ C^−1^. The relation of σ to *q*_V_ exhibits the same hysteretic behavior as for the HClO_4_ electrolyte. A fit to the averaged film stress versus the averaged specific charge yields the stress–charge coupling parameter with a value of ξ = −374 ± 2 mV. This means a larger absolute value than for HClO_4_. Figure 2d shows the respective fit for ξ.

**Figure 3:**
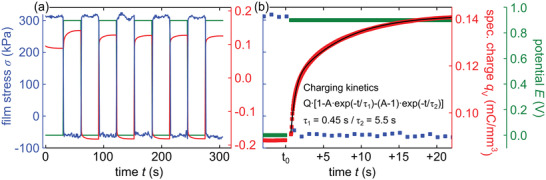
Electro‐actuation kinetics of nanoporous silicon. Step‐coulombmetry is performed in 1mol L^−1^ HClO_4_ electrolyte solution to determine the electrochemical actuation kinetics. The applied potential *E* is changed in an instant from 0.0 to 0.9 V and backward thus resembling a square potential. The thereby incorporated volume specific charge *q*_V_ and the caused film stress σ are measured versus time *t*. Whereas (a) shows the entire measurement, (b) depicts a close up of one potential increase from 0.0 V to 0.9 V at time *t*_0_. The signal of *q*_V_ is fitted by a sum of exponential functions (black).

To characterize the kinetics of the electrochemical actuation, the response of the film stress σ and the specific charge *q*_V_ to a square potential from 0.0 to 0.9 V is measured. The measurement is conducted in HClO_4_ as the electrolyte.The resulting graphs are depicted in **Figure** **3**a,b. It is shown that σ increases to its saturation level of ≈+310 kPa with a decreasing potential step, while *q*_V_ decreases to a level of −0.182 mC mm^−3^. Vice versa, σ and *q*_V_ reverse to ≈−65 kPa and 0.125 mC mm^−3^ when the potential is increased to 0.9 V.

The charging and discharging of *q*_V_ upon voltage increase and decrease is fitted by the sum of two exponential functions in the form of *Q*_ampl_[1 − *A*exp(−*t*/τ_1_) − (1 − *A*)exp(−*t*/τ_2_)], where *Q*_ampl_ and *A* denote amplitude parameters and τ_1_ and τ_2_ characteristic time constants of the two respective exponential functions. This approach has been found to appropriately approximate the charging characteristics of supercapacitor materials in response to a square potential.^[^ ^56^, ^57^ ^]^ The fit is depicted on an exemplary cycle in Figure 3b. The respective obtained quantities, averaged over the five increasing and decreasing potential steps, amount to, $\tau_{1,q_{V}\text{​},\text{decr}}=0.072\;\pm \;0.005\;s$ for the fast volume specific charge decrease, $\tau_{2,q_{V}\text{​},\text{decr}}=3.3\;\pm \;0.5\;s$ for the slow volume specific charge decrease, $\tau_{1,q_{V}\text{​},\text{incr}}=0.46\;\pm \;0.02\;s$ for the fast volume specific charge increase and $\tau_{2,q_{V}\text{​},\text{incr}}=5.7\;\pm \;0.1\;s$ for the slow volume specific charge increase. Thus, the sample is charged and discharged on two different timescales, whereby one is noticeably quicker. The change in film stress upon the instant potential increase occurs too fast for the setup's time resolution of 1s, cf. the close up in Figure 3b. Hence, the electro‐mechanical response is faster than the overall charging kinetics, on a timescale obviously smaller than 1s. Consequently, the stress response kinetics seem to be connected with the faster charge movement process. Therefore, one may speculate that the quick mechanical response is due to the fast formation of the charge layer closest to the pore wall–electrolyte interface, that is, the Stern layer, whereas the remaining, slower build‐up of the rather diffuse, Gouy–Chapman counter‐ion layer contributes negligibly to the electrocapillarity response.

An estimate for the timescale of charging $t_{c}$ can be made by assuming diffusion of ions in an ideal cylindrical pore^[^ ^58^, ^59^ ^]^

(3) $$t_{c}=\frac{2\lambda }{a}\frac{l_{\text{pore}}^{2}}{D}$$

where *a* = 3.36 nm and *ℓ*_pore_ = 630 nm denotes the pore diameter and length, respectively. λ is the Debye length and is given by λ = 0.304 nm/√(*c*_0_) = 0.304 nm for a monovalent salt diluted in water at 298K, where *c*_0_ gives the concentration of perchlorate. The equation for λ in this reduced form requires the numeric value of the concentration, which here equals 1, in units of mol L^−1^.^[^ ^47^ ^]^ The diffusion coefficient is *D* ≈ 2 × 10^−9^ m^2^ s^−1^.^[^ ^60^ ^]^ Thus, *t*_c_ ≈ 36 μs is much smaller than the measured charging timescales. Note, however, that the silicon oxide in contact with aqueous electrolytes is hydroxylated. For such hydrophilic surfaces both experiments^[^ ^61^, ^62^, ^63^, ^64^ ^]^ and computer simulations^[^ ^65^, ^66^, ^67^ ^]^ suggest the formation of highly viscous interfacial water layers of ≈1 nm thickness. In these layers the corresponding water self‐diffusivities is expected to exponentially increase toward the solid wall to values up to two orders of magnitude larger than in bulk water.^[^ ^67^ ^]^ Thus, the reduced mobility of interfacial water molecules and the corresponding reduction in the ion self‐diffusivity may explain at least partially the extremely slowed down charging kinetics observed here compared to the idealistic considerations, in particular the assumption of bulk self‐diffusivities for the confined perchlorate ions. The partially dentritic and large pore surface roughness, see also the discussion below, may increase the effective diffusion length and thus additionally contribute to a reduced charging dynamics. Still, it is worthwhile to mention that the overall charging and stress response kinetics is much faster than observed for a hybrid material of porous silicon filled by an electrically conductive polymer.^[^ ^23^ ^]^ There, the ions have to penetrate the confined polymer during the charging process, which leads to longer characteristic times around 15s for the charging and 3s for the mechanical response, respectively. Thus, the actuation scheme explored here is superior in terms of actuation kinetics.

### 2.2 Electrochemo‐Mechanical Actuation at Bulk Silicon Surfaces

**Figure 4:**
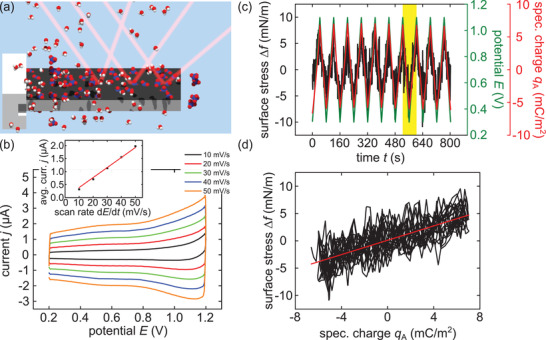
Electrochemistry and in situ laser cantilever bending experiments on bulk silicon immersed in aqueous electrolytes. a) Schematic depiction of the in situ cantilever bending measurement on a bulk silicon sample (light gray) with a silicon oxide layer on top (dark gray) in an aqueous (red, white molecules) perchloric acid (blue, red molecules) electrolyte solution. The sample is clamped on the left by poly(tetrafluorethylene) (PTFE). The sample that is in contact with the electrolyte solution has a length of 4.28 cm and a width of 1.49 cm. b) The graph shows exemplary CV measurements of a bulk silicon sample in 1mol L^−1^ HClO_4_ electrolyte. The current *j* is plotted against the applied potential *E*. Five measurements with scan rates from 10 to 50 mV s^−1^ are shown. The inset depicts values for the current *j*, averaged in the potential range of 0.6–0.8 V, plotted against increasing potential scan rates. The red line gives a linear regression of the data and yields a capacitance of *c* = 38.51 ± 0.08 μF. c) Measurement of the change in surface stress Δ*f* in a 1mol L^−1^ HClO_4_ solution under a changing potential *E* with a scan rate of 20 mV s^−1^ in the potential region of 0.3–1.1 V. The surface area specific capacitance *q*_A_ is depicted as well. d) Change in surface stress Δ*f* versus the specific charge *q*_A_. To independently determine the surface stress–charge coefficient ς a linear regression is used. It yields ς = +0.657 ± 0.007 V.

To gain mechanistic and detailed quantitative insights on the actuation of porous silicon, also experiments on the planar, nonporous silicon surface are performed. A piece of bulk silicon is immersed into an electrolyte solution of 1 m HClO_4_. Thereby, the area of the sample in contact with the electrolyte amounts to 24.346 cm^2^. To render the measurements performed on bulk silicon comparable to porous silicon, the samples are treated in the same manner and the surface of bulk silicon is oxidized by applying a constant potential of 1.2 V for 15 h. The reasons for applying a constant potential to the sample beforehand lie in the oxidative nature of the silicon surface as discussed in great detail above and in the Supporting Information. The recorded current is decreasing to 92 nA. Subsequently, CV measurements are performed, depicted in **Figure** **4**b. The potential range is 0.2–1.2 V with scan rates from 10 to 50 mV s^−1^. A characteristic capacitive charging is apparent for all depicted scan rates. Albeit for larger potentials from 0.8 V on for the fastest scan rate of 50 mV s^−1^ a slight increase toward the upper vertex of the CV becomes apparent. No distinct oxidation or reduction peaks on the up‐ and down‐sweep, respectively, are noticeable.

The plot to determine the capacitance *c* is depicted in the inset of Figure 4b. The constant current for each scan rate is gained by averaging the absolute current values between 0.6–0.8 V for both sweep directions. A linear dependence is visible up to scan rates of 50 mV s^−1^. The linear regression yields a capacitance of *c* = 38.51 ± 0.08 μF. Additionally, the roughness of the oxidized bulk silicon surface has been determined with an atomic force microscope. The roughness increases the apparent surface by a factor of 1.00075. Therefore, the bulk silicon sample has a surface specific capacitance of c* = 1.579 ± 0.003 μF cm^−2^.

Next, the measurement of a bulk silicon sample in the cantilever bending setup is analyzed. CV measurements with scan rates of 10, 20, 30, 40, 50, and 100 mV s^−1^ are performed. The resulting measurements all exhibit the same capacitive characteristics already seen for the bulk silicon sample in Figure 4b, and are shown in Figure S5a, Supporting Information. The linear regression of the averaged currents plotted versus the respective scan rate, as shown in Figure S5b, Supporting Information, yields a capacitance of *c* = 10.07 ± 0.08 μF. Therefore, the surface area, that is in contact with the electrolyte solution inside the cell of the cantilever bending setup amounts to *c*/c* = 10.07 μF/1.579 μF cm^−2^ = 6.38 ± 0.05 cm^2^. The respective measurement of the surface stress for the CV measurement with a scan rate of 20 mV s^−1^ is depicted in Figure 4. The charge accumulation related curvature change Δκ is already converted to a change in surface stress Δ*f* according to Equation (10). Figure 4c shows an identifiable surface stress change of the bulk silicon sample. Hence, to highlight this point, the laser cantilever experiments provide the sensitivity to measure an electrochemical actuation in a bulk silicon sample. The measurement exhibits a change in surface stress Δ*f* that is well in phase with the potential *E* and the accumulated charge per sample surface *q*_A_. The plot of Δ*f* versus *q*_A_ in Figure 4d further emphasizes this linear relation. Here, a linear regression of the change in surface stress Δ*f* versus accumulated surface specific charge *q*_A_ yields the electrocapillarity coupling parameter ς with a value of ς = +0.657 ± 0.007 V. Overall, this independent measurement method yields a dependable value for the surface stress–charge coefficient ς. It represents a fundamental parameter for the material and can be utilized in the analysis of the actuation properties of the porous silicon samples or for other experiments where the electrochemistry of silicon is of importance.

## 3 Discussion

The microscopic cause of the electrochemical actuation phenomenon is rooted in surface stress, that is caused by the charging of the surface. Through our measurements it becomes apparent that this relation is also valid for porous silicon.

The dependence of macroscopic dimensional change δ*l*/*l* on surface stress *f* can be described by a model that assumes a porous material in the form of smooth, cylindrical, parallel oriented, and hexagonally arranged pores, that stretch from top to bottom.^[^ ^68^ ^]^ As the porous structure of porous silicon suits these assumptions, the model is thus appropriate to be utilized. The change in length δ*l*/*l* is then^[^ ^68^ ^]^

(4) $$\delta l/l=\delta \varepsilon =-\frac{\alpha f}{3K}\frac{\left(1-\nu \right)}{\left(1-2\nu \right)}$$

where α = 1 − Φ denotes the solid volume fraction (with porosity Φ = 0.357, see Supporting Information), *K* = 97.8 GPa, the bulk modulus of silicon, and ν = 0.064, Poisson's ratio for silicon in the (110) direction.^[^ ^69^ ^]^ Interestingly, the relation for δ*l*/*l* is similar to that of nanoporous gold, albeit their very different porous morphologies.^[^ ^68^ ^]^ Based on Equations (4) and (2) the already mentioned strain–charge coupling parameter A* reads

(5) $$A^{*}=\frac{d\varepsilon }{dq_{V}}=-\frac{\alpha \varsigma }{3K}\frac{\left(1-\nu \right)}{\left(1-2\nu \right)}$$

The value that has been determined for ς in this study suggests A* = −0.0015 mm^3^ C^−1^. In a next step this value, deduced from the measurement of the bulk silicon sample, is compared with the measurement of the porous silicon sample. Hence, the characteristic film stress–charge coupling parameter ξ is transformed into a strain–charge coupling parameter. The necessary equation reads

(6) $$A^{*}=\frac{\varepsilon_{\left|\right|}}{q_{V}}=\frac{1}{E_{\left|\right|}}\xi$$

where ε_||_ denotes the strain and *E*_||_ the Young's modulus in the plane of the porous silicon layer, and ξ = −296 mV for the measurement with perchloric acid. The crystal symmetry of the porous silicon layer can be approximated as in‐plane, transverse isotropic, where the pores break the cubic crystallographic symmetry of the silicon pore walls, as a recent study finds.^[^ ^70^ ^]^ Thus, *E*_||_ can be determined by

(7) $$\frac{1}{E_{\left|\right|}}=\frac{c_{33}}{-2c_{13}+c_{33}(c_{11}+c_{12})}\approx \frac{1}{28.07\;\text{GPa}}$$

with *c*_i_ being the elastic coefficients^[^ ^70^, ^71^ ^]^ and approximating the difficult to measure coefficient *c*_12_ as *c*_12_ ≤ *c*_13_. Equation (6) then yields A* = −0.011 mm^3^ C^−1^ as a lower absolute limit, which corresponds to a strain amplitude by the porous silicon layer of $A*q_{V_{\text{avg}}}=1.69\pm 0.04\times 10^{-3}\%$ or ≈50.5 μm for the sample of length *l*. The value A* = −0.011 mm^3^ C^−1^ is about one order of magnitude larger than A* = −0.0015 mm^3^ C^−1^, the value obtained by Equation (5). A reason for the present deviation could be due to the morphology of the porous structure of the here presented porous silicon. They deviate a lot from the idealized straight, round channel with a smooth surface, that underlies the theoretical calculations in ref. [68]. The SEM image depicted in Figure S1, Supporting Information gives a distinct impression of the porous morphology. Thereby, the main pores are slightly meandering. Furthermore, smaller pores branch off the main pores. Thus, the pore walls are far from resembling their smooth equivalent assumed in the theoretical study. These side pores can be considered as an extreme increase in roughness. For silicon, surprisingly, such an increase in roughness even results in an enhanced electrochemo‐mechanical coupling of the surface stress‐induced actuation. This is in stark contrast to metals with large Poisson ratio such as copper or gold.^[^ ^72^ ^]^ For nanoporous gold, a well‐investigated material in the regard of electrochemical actuation, a strain–charge coupling parameter of A* = 0.0475 ± 0.0010 mm^3^ C^−1^ has been found.^[^ ^46^ ^]^ Thus, the absolute value of A* = −0.011 mm^3^ C^−1^ obtained in this study for porous silicon lies on the same order of magnitude, albeit being ≈4 times smaller. Aside from the entirely different porous structure of nanoporous gold, a distinct difference between the materials is their conductive properties. Whereas gold is an excellent conductor, silicon is semiconducting. In gold, the electrons that counter the ionic charge carriers which accumulate in the Helmholtz layer on the electrolyte side of the interface, assemble in an Å‐thin layer beneath the interface. Conversely, when an oxidized silicon wall is in contact with an electrolyte solution, a space charge region beneath the silicon‐oxide interface emerges. The charge carriers, holes in the case of p‐doped silicon, are distributed over the width of the space charge region.^[^ ^48^ ^]^ The width *w* of the space charge region can be calculated by

(8) $$w=\sqrt{\frac{2\varepsilon_{s}}{eN_{D}}\left(V_{\text{fb}}+\frac{k_{B}T}{e}\right)}$$

where ε_s_ = ε_0_ε_r_, with ε_0_ and ε_r_ denoting the vacuum and relative permittivity.^[^ ^48^ ^]^ *N*_D_ is the doping concentration, *V*_fb_ the flat band potential, where the bands at the electrolyte interface are not bend but straight and *k*_B_ and *T* denote the Boltzmann constant and the temperature. It can be readily seen, that the width of the space charge region foremost depends on the doping concentration *N*_D_ and the flat band potential *V*_fb_. With the assumption of a pure silicon interface, thus ε_r_ = 11.68, a doping concentration of *N*_D_ = 5 × 10^18^ cm^−3^ and a flat band potential of 0.4 V, the space charge region has a width of *w* = 9.8 nm.^[^ ^73^, ^74^, ^75^ ^]^ Thus, the charge carriers that cause the surface stress are distributed in a layer about one order of magnitude thicker than in gold. Hence, the actuation response upon an equal accumulated charge is possibly smaller, which is, what is observed here.

Overall in this study, we showed the versatile nature of porous silicon with regard to possible electrochemical actuation applications. The investigation of the characteristic electrochemical behavior in acidic and salt solutions and the thereby induced surface stress related actuation lead to a deeper understanding of porous silicon. Furthermore, surprisingly we find an inversely proportional relation of the actuation on potential and charge. For the first time, the vital, fundamental surface stress–charge coupling parameter ς could be determined for silicon surfaces.

Note that for a reliable stress analysis a thin porous layer was employed on a thick bulk layer of silicon in our actuation experiments. If large strains are the goal one could easily achieve an amplification of the electro‐mechanical response by reducing the thickness or by entirely removing of the bulk silicon layer. Similarly as it has been demonstrated for liquid‐infused porous structures with regard to confinement‐controlled phase selection,^[^ ^7^, ^76^ ^]^ adaptable surface wetting,^[^ ^77^, ^78^ ^]^ topography,^[^ ^79^ ^]^ photonic,^[^ ^80^, ^81^ ^]^ and mechanical properties^[^ ^24^, ^82^ ^]^ our study indicates that electrolyte‐infused nanoporous solids allow for a quite simple fabrication of materials with integrated electro‐actuorics.

Mechanical stress in biological systems affects growth kinetics, form, and function of many living tissues.^[^ ^83^, ^84^, ^85^ ^]^ Most prominently, mechanical stimulation results in altered cell morphologies, changes in cell signaling, and gene transcription via activation of mechanoreceptors such as piezochannels.^[^ ^86^ ^]^ The mechanical force capabilities of nanoporous silicon demonstrated here along with its biocompatibility could therefore be employed in (bio‐)medical surfaces and implants for electrically controlled manipulation of tissues and mechanotransduction, that is, conversion of mechanical forces to biochemical signaling. It has also been demonstrated that electrocapillary coupling in nanoporous media can be employed in the inverse direction, that is, for the transformation of mechanical stress directly into electrical signals.^[^ ^46^ ^]^ Thus, one can envision that on‐chip integrated nanoporous silicon can be employed to sense stresses in vivo or in electrolytic environments in general, which is also a particular challenge in structural health monitoring and in the biomaterials sciences and technologies.^[^ ^84^, ^85^ ^]^

Finally, our study is also a fine example, how the combination of soft and hard matter opens up the possibility of using multi‐physical couplings in geometrical confinement from the nano‐ via the meso‐ to the macroscale to design robust hybrid materials with integrated functionality, as it can be found in many biological composites.^[^ ^87^, ^88^ ^]^

## 4 Experimental Section

**Figure 5:**
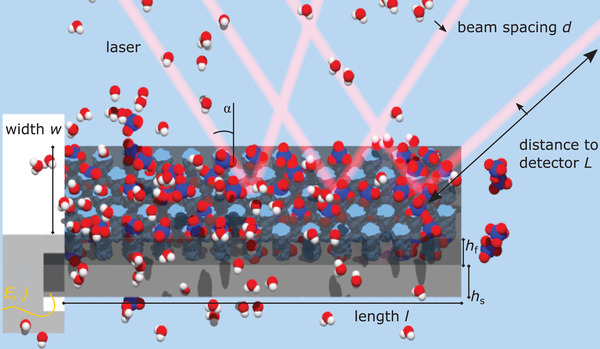
Schematic depiction of the sample geometry and measurement setup. The sample consists of a porous layer (dark gray) attached to a remaining bulk silicon layer on the bottom (light gray). The pores are filled with electrolyte solution indicated by the blue color and the HClO_4_ (blue, red) and H_2_O (red, white) molecules. The dimensions of the fabricated sample are length *l*, width *w*, and the thickness of the substrate *h*_s_, and the porous silicon film *h*_f_, respectively. Moreover, the figure depicts the experimental cantilever bending setup. The sample acts as the working electrode. It is contacted by an aluminum contact and an attached gold wire on the left side to apply potential *E* and measure current *j*. The potential *E* in the electrolyte solution can be changed, so that the ${\text{ClO}}_{4}^{-}$ anions are accumulated on the bulk silicon surface, or the porous silicon pore surface, respectively, or so that the anions are expelled. The result is an induced surface stress which leads to a contraction of the sample followed by its subsequent expansion. Since the underlying bulk silicon substrate clamps the film, the film is stressed which leads to a curvature of the substrate in return. A laser beam is split into an array and gets reflected into a CCD detector, to measure the curvature κ of the sample. The laser here is aimed at the top side of the sample for illustrating purposes, whereas in the actual experiment it is reflected off the backside. The laser array is also aimed at the fare end of the clamped side, to avoid an influence on the bending by the clamping. The distance between sample and detector is denoted by *L*, the incident angle of the laser beam array by α and the spacing between the individual laser beams hitting the detector by *d*. The sample is clamped on the left by PTFE. The whole setups is housed in an electrochemical cell, that is fabricated out of PTFE as well and sealed on the top with an optically transparent glass plate.

Two types of sample were investigated in this study—bulk silicon and porous silicon. The base material for the fabrication of both types of samples was p‐doped single‐crystalline silicon. The supplier (Si‐Mat Silicon Materials GmbH) gave a thickness of 100 ± 10 μm for the wafers, a (100) orientation and a resistivity of 0.01−0.02 Ωcm. Both back‐ and frontside of the wafer were polished. The electrochemo‐mechanical actuation properties of bulk silicon, particularly the response of surface stress *f* to accumulated charge *q*, that is, the electrocapillarity coupling parameter ς, were investigated on this same base material. The sample for such a measurement was prepared in the following way. A p‐doped wafer was oxidized in an oven at 850 °C for 16 h, with a heating and cooling ramp of 200 °C h^−1^, to create a thick, nonconductive insulation layer of silicon dioxide.^[^ ^26^ ^]^ The thickness of this layer was determined to be 96.3 nm by ellipsometry. Subsequently, the insulation layer was removed on the frontside by a hydrofluoric acid (HF) dip in 10% aqueous HF solution for 5 min. The actual sample was then prepared from the fabricated silicon material by cleaving.^[^ ^6^ ^]^ The sample was shaped rectangularly with a long side of 5.37 cm and a short side of 1.49 cm. The surface area which was in contact to the solution, though, was smaller. Not all of the sample was immersed into the electrolyte solution, as the top of the sample was clamped by PTFE. The surface area in contact to the electrolyte is precisely determined in Section 2.2 and amounted to 6.38 cm^2^. Porous silicon was prepared by etching the frontside of the silicon wafer with the prepared backside insulation layer in an electrochemical procedure with HF. The wafer was electrically contacted by aluminum foil and mounted into an electrochemical cell. The cell was made from PTFE, to safely handle HF. A fluoro‐elastomer‐made O‐ring with an inner radius of 3.30 cm sealed the contact between wafer and cell. First, the insulation layer was removed with the above‐described HF‐dip. Then the electrolytic solution was exchanged and the cell was filled with a 2:3 volumetric mixture of HF (48%, Merck Emsure) and ethanol (absolute, Merck Emsure). The HF electrolyte was allowed to equilibrate for 5 min. A platinum counter electrode (CE) was inserted into the cell from the top. It acted as the cathode, whereas the silicon was the anode. The accordingly called anodization or etching process started by applying a constant current of 428 mA which equaled a current density of 12.5 mA cm^−2^. After 1 min the current was switched off and the HF solution was removed from the cell. In a final step the resulting porous silicon was rinsed three times with distilled water, demounted from the cell and dried at ambient conditions for 3 h. The actual sample was prepared from the fabricated porous silicon material equally to the bulk silicon sample by cleaving. The sample had a length of *l* = 2.990 ± 0.002 cm and a width of *w* = 1.796 ± 0.002 cm. To stress this point, the porous layer was not detached in a so‐called electropolishing step from the remaining bulk silicon. Rather the porous layer stayed attached to the bulk silicon. **Figure** **5** gives a sketch of the sample and its dimensions. TEM was used to record a microscopy image of a cross‐section of the porous silicon (FEI Helios G3 UC). To measure the thickness of the top and bottom porous layer, a profile was recorded with a canning electron microscope (Zeiss Leo 1530). Further properties of the resulting porous silicon as mean pore diameter, porosity and internal surface area were characterized in a nitrogen sorption isotherm setup (Quantachrome autosorb iQ). A simple and sensitive experiment for the measurement of the actuation properties represents an in situ cantilever bending investigation, described in great detail by Smetanin et al.^[^ ^42^ ^]^ The respective sample, bulk or porous silicon, was installed into the in situ cantilever bending setup. It was electrically contacted at the top end by an aluminum contact and an attached gold wire. As the sample was exposed to an acidic solution, the contact needed to be protected from corrosion. A scheme of the setup can be found in Figure 5. The sample was immersed in one of the two aqueous electrolytic solutions—perchloric acid (HClO_4_) with a concentration of 1 mol L^−1^ or a sodium chloride (NaCl) solution with a concentration of 154 mol L^−1^ (isotonic saline solution). HClO_4_ was prepared from 70% perchloric acid (Merck Suprapur) and deionized water (18.2 MΩ). Deionized water was used as well to solve NaCl (Merck Suprapur). Before a change of electrolyte solution, the sample was rinsed three times with deionized water and dried at ambient conditions for 3 h. The surface area of the porous silicon sample, which was in contact to the solution amounted to 5.37 cm^2^. A carbon cloth CE and a reversible hydrogen reference Electrode (RE, Gaskatel HydroFlex) were inserted into the chosen solution. All electrochemical potentials in this work are denoted versus the standard hydrogen electrode (SHE). The electrodes and the sample were connected to a potentiostat (Metrohm‐Autolab PGSTAT 30) and thus electrochemical measurements can be performed.

A potential *E* was applied between sample and CE, which was measured and controlled via the RE by the potentiostat. Additionally, the potentiostat measured the current *j* that flowed between sample and CE and provided the accordingly consumed charge *Q* as well. The applied potential can be changed with a linearly varying slope between a lower and higher reversal potential, a so‐called cyclic voltammetry measurement. By these means, certain electrochemical characteristics as the capacitance of the sample or the presence of electrochemical reactions with an electron exchange, that is, reduction or oxidation reactions, can be inferred. The rate d*E*/d*t* with which the potential was varied is called the sweep or scan rate. The theoretical potential range, in which a CV can be conducted in aqueous solutions, lies in between 0 and 1.223 V. Below, a reduction of the soluble, that is, water, occurs and hydrogen gas forms. Above, at potential values higher than 1.223 V, water itself starts to be oxidized and oxygen gas emerges, although in practice different electrolyte solutions have an overpotential.

Another type of measurement used was an instant change of a lower potential to a higher value and vice versa in a step‐like fashion, also known as step coulombmetry. After changing the potential it was subsequently kept constant for a period of time, resembling a square potential. In this way, it was possible to determine the kinetics of the electrochemical processes and the accompanying change in actuation. Furthermore, the characteristic time constant of the evolution in charge and the cantilever bending signal upon this instant change in potential can be determined. Lastly, the potential can also be kept constant at a single value while the current is recorded.

Altogether, when the potential was changed, the solution's ions were accumulated on the bulk silicon surface or on the surface of the porous silicon layer, respectively. Thus, charge carriers were not accumulated on and dispersed off the wafer's insulated backside but solely on the frontside surface. The accumulated charge thereby changed accordingly to the surface stress. This lead to a bending of the wafer. It can be detected and measured by a laser setup, where a laser beam array was reflected off the backside of the cantilever‐like sample and detected in a CCD sensor. The setup (Multi‐Optical Stress Sensor, k‐Space Associates, Inc.^[^ ^89^, ^90^ ^]^) is described in great detail in ref. [42]. To minimize the impact of vibrations the setups were installed on a vibration isolated table. The setup was surrounded by isolation housing, to reduced vibrations by air flow. Thermal drift was reduced by air conditioning the room at 21 °C. Ref. [53] determined a resolution of bending radius of 250km for this specific setup, which was sufficient for the measurements conducted here. The time resolution was 1 s. The spot spacing Δ*d* of the array of laser beams changed with the curvature of the sample and thus the evolving surface stress. Thereby, the resulting resolved curvature changed parallel to the long axis of the wafer Δκ was the relevant parameter and was determined by^[^ ^42^ ^]^

(9) $$\Delta \kappa =\frac{\Delta d}{d_{0}}\frac{\cos(\alpha )}{2Ln}$$

where *d*_0_ is the unimpeded spacing averaged over 1 min, α = 4° the incident angle of the beam array, *L* = 110 cm the distance from the laser to the CCD camera, and *n* = 1.33 the refractive index of water as a good approximation for the diluted aqueous electrolyte solution. In case of the bulk silicon, the curvature change was a result of a change in the surface stress and was related to the curvature via Stoney's equation^[^ ^42^, ^91^, ^92^, ^93^ ^]^

(10) $$\Delta f=-\frac{1}{6}Mh^{2}\Delta \kappa$$

where *M* = 180 GPa is the biaxial modulus^[^ ^69^ ^]^ and *h* = 106.71 ± 1.03 μm is the thickness of the bulk silicon sample. For the porous silicon sample a film stress σ evolved in the porous silicon film and assuming it was isotropic in the film plane, Stoney's equation related it to Δκ via

(11) $$\Delta \sigma =-\frac{1}{6}M\frac{h_{S}^{2}}{h_{f}}\Delta \kappa$$

Thereby, *h*_S_ and *h*_f_ denote the thickness of the substrate and the film, respectively, and are given in the results section. It is important to notice that the analysis of the film stress via Stoney's equation (Equation (11)) only demands knowledge of the mechanics of the substrate and not the film. This assumption that the contribution of the film to the overall bending stiffness can be ignored, requires that the film's thickness *h*_f_ does not greatly exceed the substrates’ thickness by a factor of 2 × 10^−3^.^[^ ^94^ ^]^ Here, *h*_f_/*h*_S_ ≈ 5.8 × 10^−3^ was well within that range.

## Conflict of Interest

The authors declare no conflict of interest.

## Author Contributions

M.B. and P.H. conceived the experiments. M.B. performed the material synthesis and the actuation measurements. M.B. and P.H. evaluated the data. M.B. and P.H. wrote and proofread the manuscript.

## Supporting information

Supporting Information

## Data Availability

The raw data of the electrochemical actuation experiments are openly available at TORE (https://tore.tuhh.de/), the Open Research Repository of Hamburg University of Technology, at DOI: https://doi.org/10.15480/336.3790.
