# Supplementary material for: Wafer‐Scale Electroactive Nanoporous Silicon: Large and Fully Reversible Electrochemo‐Mechanical Actuation in Aqueous Electrolytes
Source: Adv Mater. 2021 Oct 22;34(1):2105923. doi: 10.1002/adma.202105923 (PMC11468870; doi:10.1002/adma.202105923)
Supplement: Supplementary file 1 — Supporting Information [file ADMA-34-2105923-s001.pdf]

ADVANCED MATERIALS

Supporting Information

for *Adv. Mater.,* DOI: 10.1002/adma.202105923

Wafer-Scale Electroactive Nanoporous Silicon: Large and Fully Reversible Electrochemo-Mechanical Actuation in Aqueous Electrolytes

Manuel Brinker and Patrick Huber**

Wafer-Scale Electroactive Nanoporous Silicon: Observation of Large and Fully Reversible Electrochemo-Mechanical Actuation in Aqueous Electrolytes - Supporting Information

Manuel Brinker Patrick Huber**

Manuel Brinker, Prof. Dr. Patrick Huber
Address: Hamburg University of Technology, Institute for Materials and X-Ray Physics, 21073 Hamburg; Deutsches Elektronen-Synchrotron DESY, Center for X-Ray and Nano Science, 22607 Hamburg, and University of Hamburg, Centre for Hybrid Nanostructures CHyN, 22607 Hamburg, Germany.
*Email Addresses: manuel.brinker@tuhh.de, patrick.huber@tuhh.de

Keywords: *nanoporous media, electrochemical characterization, actuorics, cyclic voltammetry, laser cantilever bending*

1 Oxidation of Nanoporous Silicon

The initial electrochemical charaterization of the as fabricated nanoporous silicon sample comprises two consecutive CV cycles conducted with a scan rate of $10\,\mathrm{mVs^{-1}}$ in the potential range of 0 to 1.2 V. It is depicted in Figure S3(a). It is visible that the current increases with an increasing potential. When the upper vertex at 1.2 V is reached and the scan direction is reversed, the current decreases from its peak value of 4.367 mA, but with a larger slope. Thus, the current does not follow the same course as on the up-sweep and an area is encompassed by the up- and down-sweep of the current. Hence, the measurement indicates that a capacitive charging is present. But the current due to the capacitive charging is eminently superimposed by a current that is of oxidative nature. This inherent current increases in an exponential way with the potential. The peak current at the upper vertex successively decreases from the first to the next cycle. These characteristics of the CV can be attributed to an oxidation of the porous silicon walls to silicon dioxide by the applied potential in the aqueous environment.[1, 2, 3] The sample surface is hydrogen terminated directly after fabrication, meaning the silicon surface bonds are occupied by hydrogen atoms. However, since the sample is not handled in a controlled ambient atmosphere, a native oxide layer forms.[4, 5] Through an increasing applied potential the silicon pore walls are further oxidized. The surface area that is oxidized in subsequent cycles, decreases. Hence, the current grows smaller from cycle to cycle, displaying also the electrochemically irreversible character of this oxidative process. To foreground the capacitive current and suppress the oxidative current, the silicon oxidation has to be drawn to a close. Therefore, a constant potential of 1.2 V is applied for 20 hours. The resulting current is shown in Figure S3(b). It is clearly visible, that the current drastically decreases, to a value of 3.62 μA. This oxidative process already results in a distinct increase of the film stress σ in the porous silicon layer. It increases to an asymptotic value of 42.06 MPa. This significant increase in film stress is likely caused due to a mismatch of the lattice constants of the remaining silicon in the porous silicon pore walls and the adjacent electrochemically fabricated silicon dioxide layer on the pore wall.[6]
A set of CV measurements recorded following the applied constant potential with a scan rate of $10\,\mathrm{mVs^{-1}}$ is depicted in Figure S3(c). Firstly, it is apparent that the large currents that have occurred with increasing potentials are vastly reduced. So, the current value at the upper vertex at 1.2 V declines to 8.07 μA. Through the applied constant potential the features of the CV measurements are more pronounced. It is visible, that the potential region mainly exhibits currents due to a capacitive charging of the internal porous silicon surface area. That is to say, a current slope due to a Faradaic reaction is mostly absent and only visible from about 1.0 V on to the vertex of 1.2V. On the down-sweep from 0.4 V to 0 V, the oxygen reduction reaction comes into effect. Thereby, solved oxygen is progressively reduced in the potential range from 0.4 V to 0 V, which generates the negative current slope.[7] Hence, this current slightly superimposes the currents generated by the ordinary electrochemical charging of the sample.
The aim is to further investigate the process of the oxidizing step on porous silicon. Therefore, an essentially identical porous silicon layer with a porous layer thickness of 25 μm is fabricated and subsequently oxidized.

The potential of 1.2 V is applied for 42 hours in 1 M $HClO_4$. The current likewise drastically decreases to a value of 22 μA. Additionally, the change in weight from the unoxidized state to the oxidized one is determined to $\Delta m = +6.9$ mg. The resulting nitrogen sorption isotherme is depicted in the supplementary materials in Figure S6. The sorption isotherm exhibits the characteristic hysteresis between ad- and desorption. However, it is visible, that the hysteresis is more pronounced, as the capillary condensation sets in at a lower relative vapor pressure compared to the unoxidized porous silicon. The evaluation with the BJH model, depicted in the inset, yields a bimodally distributed radius with the main peak at $r = 2.67$ nm and a second smaller peak at $r = 1.97$ nm, that is more noticeable than the shoulder in the unoxidized material. Thus, the mean radius is reduced by approximately $\Delta r = 0.69$ nm compared to the before measured $r = 3.36$ nm. The reduction in pore diameter is well known in terms of a thermal oxidation of porous silicon.[8, 9] Additionally, the porosity decreases to a value of $\Phi = 35.7\,\%$. Applying the BET model results in a value for the inner surface area of $A_{\text{oxid.}} = 2.36\,\text{m}^2$ which is a significant decrease from an initial value of $A_{\text{init.}} = 4.755\,\text{m}^2$ for an unoxidized sample. For the smaller, p-doped electrochemical actuation sample, that was measured in the cantilever bending setup, an oxidized inner surface area of $A_{\text{sample,oxid.}} = 712\,\text{cm}^2$ is obtained.
The anodic oxidation reaction which is responsible for the process reads as follows,[3]

$$\text{Si} + 2\text{H}_2\text{O} + nh \rightarrow \text{SiO}_2 + 4\text{H}^+ + (4-n)\text{e} \qquad (n \leq 4), \tag{1}$$

where h is a hole charge carrier, the majority type in p-doped silicon, and n is the consumed number of holes per reaction, which equals 4 for a full conversion. The full reaction path is still under discussion and not fully understood.[3] A possible synthesis path involves the creation of hydroxide.[10] This reaction may be responsible for a feature noticeable in the electrochemical analysis of the porous silicon sample. In the oxidized state, the CV with a scan rate of $200\,\text{mVs}^{-1}$, depicted in Figure 1(c), features a small, but noticeable, peak in the up-sweep at 0.553 V. Additionally, another peak becomes visible in the down-sweep at 0.431 V. In total, the two peaks might resemble a redox couple of an oxidation and reduction process. The OH-groups may be ad- and desorped in a chemisorption process, which has been reported before and measured in a similar manner.[11]
The oxidation of the porous silicon consumes a total charge of 65.175 C for the time the potential is applied. Thus, assuming $n = 4$, the reaction forms $0.169 \cdot 10^{-3}$ moles of silicon dioxide, which equals 10.15 mg. This value is in quite good agreement with the measured mass difference of $\Delta m = 6.9$ mg, if possible side reactions that consume charge are considered. Moreover, a value for the created volume of silicon dioxide can be inferred by Δm and by assuming a density of chemical silicon dioxide of $\rho = 2.2\,\text{g/cm}^3$.[5] This results in a volume of $V_{\text{SiO}_2} = 0.003142\,\text{cm}^3$. The thickness of the SiO_2 layer is then $d_{\text{SiO}_2} = V_{\text{SiO}_2}/A_{\text{init.}} = 0.66$ nm, which excellently fits the mean pore diameter reduction $\Delta r = 0.69$ nm. Hence, a homogeneously distributed creation of silicon dioxide throughout the porous silicon sample is found to be the reason for the reduction in the inner surface area and the mean pore radius.
Furthermore, it is possible to compare the physical surface area, determined by nitrogen sorption isothermes, to the surface area that is addressed in the CV measurements. A value for this surface area can be extracted through the determination of the surface specific capacitance c^* of the electrochemically oxidized bulk silicon surface, conducted in this study. Thus, the sample has an electrochemical active surface area, i.e. the surface area of the porous silicon that accumulates charge, when polarized, of $c/c^* = 0.466\text{mF}/1.579\,\mu\text{Fcm}^{-2} = 295.1\,\text{cm}^2$, which corresponds to $81.2\,\text{m}^2\text{g}^{-1}$ normalized to the mass of the porous silicon layer. The electrochemical active surface area translates to $295.1\,\text{cm}^2/A_{\text{sample,oxid.}} = 41.4\,\%$ of the internal surface area determined by the BET analysis. Hence, the electrochemical active surface area is smaller than the physical surface area. The discrepancy of these surface areas might have different reasons. During the oxidation process thin parts of the porous silicon pore walls are possibly fully oxidized, so that they are electrically insulating. Thus, these parts would no longer contribute to a capacitive charging and the electrochemically active surface area would be reduced. The reduction due to this effect can be as large as 70 % of the initial surface of anodically oxidized porous silicon.[1] Another explanation might lie in the small dendritic side pores, that branch off from main pores, as can be also seen in the SEM micrograph depicted in Figure S1. These small pores might not contribute to the electrochemically active- but to the physical surface area.

References

[1] A. Bsiesy, F. Gaspard, R. Herino, M. Ligeon, F. Muller, J. Oberlin, *Journal of the Electrochemical Society* **1991**, *138*, 11 3450.

[2] F. Gaspard, A. Halimaoui, G. Sarrabayrouse, *Revue de Physique Appliquée* **1987**, *22*, 1 65.

[3] X. G. Zhang, *Electrochemistry of Silicon and its Oxide*, Springer Science & Business Media, **2007**.

[4] D. Gräf, M. Grundner, R. Schulz, L. Mühlhoff, *Journal of Applied Physics* **1990**, *68*, 10 5155.

[5] V. Lehmann, *Electrochemistry of silicon: instrumentation, science, materials and applications*, Wiley-VCH, Weinheim, **2002**.

[6] D. Buttard, D. Bellet, G. Dolino, *Journal of applied physics* **1996**, *79*, 10 8060.

[7] B. Roschning, J. Weissmueller, *Electrochimica Acta* **2019**, *318* 504 .

[8] R. Herino, A. Perio, K. Barla, G. Bomchil, *Materials Letters* **1984**, *2*, 6 519.

[9] J. R. Dorvee, A. M. Derfus, S. N. Bhatia, M. J. Sailor, *Nature materials* **2004**, *3*, 12 896.

[10] H. Lewerenz, *Electrochimica Acta* **1992**, *37*, 5 847.

[11] A. Muñoz, A. Moehring, M. Lohrengel, *Electrochimica acta* **2002**, *47*, 17 2751.

[12] R. Herino, G. Bomchil, K. Barla, C. Bertrand, J. L. Ginoux, *Journal of the Electrochemical Society* **1987**, *134*, 8 1994.

[13] S. Brunauer, P. H. Emmett, E. Teller, *Journal of the American Chemical Society* **1938**, *60*, 2 309.

[14] E. P. Barrett, L. G. Joyner, P. P. Halenda, *Journal of the American Chemical Society* **1951**, *73*, 1 373.

2 Supplementary Figures

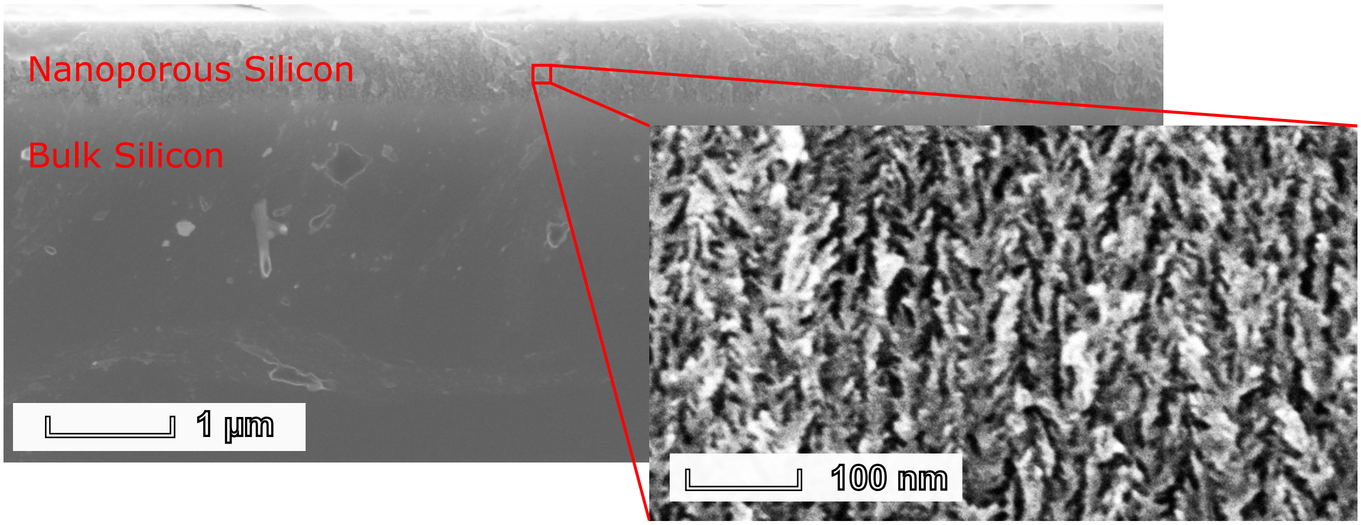

Figure S1: **Sideview of nanoporous silicon.** SEM micrograph of the nanoporous silicon profile. Visible are the pores with the etch direction from the top to the bottom. The inset shows a closeup of the nanoporous silicon structure.

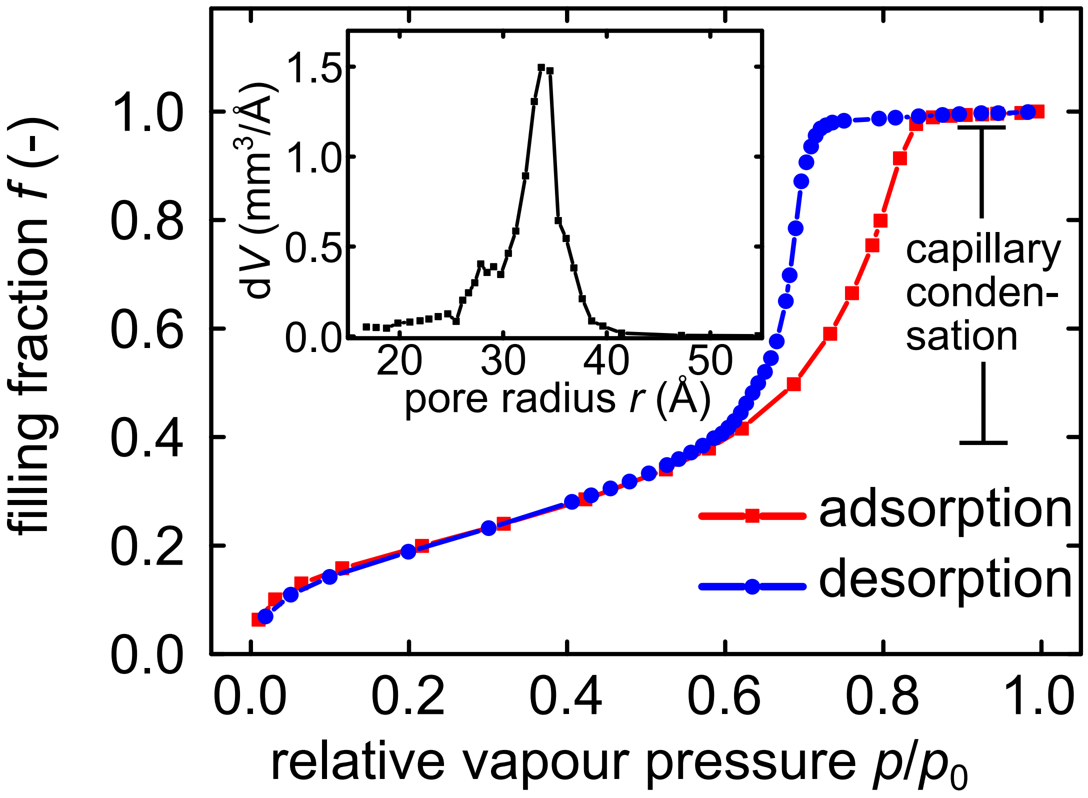

Figure S2: **Nitrogen sorption isotherm of as fabricated nanoporous silicon.** Nitrogen sorption isotherm at $T = 77\,\mathrm{K}$ recorded for as fabricated porous silicon. The volume filling fraction f is plotted against the relative vapor pressure p/p_0. The isotherm exhibits the typical characteristics of such a measurement on nanoporous silicon.[12] By increasing the relative vapor pressure p/p_0 first single layers of nitrogen absorb on the internal surface area of the porous silicon and the filling fraction increases approximately linearly up to a value of $p/p_0 = 0.7$. At $p/p_0 = 0.7$ capillary condensation sets in and menisci form in the pores. With an increasing relative vapour pressure these menisci are driven out to the end of the pores. Lastly, the sample is saturated and the filling fraction reaches 1. Subsequently, the pressure is reduced, p/p_0 decreases and the nitrogen desorbs from the menisci until the menisci vanish. Thereby, the characteristic hysteresis of the sorption isothermes is visible.
The BET model is used to determine the actual internal surface area of the porous silicon.[13] The result equals $A_{\mathrm{por}} = 4.003\,\mathrm{m}^2$ for a material volume of $0.0165\,\mathrm{cm}^3$. The BJH model is used on the desorption branch of the sorption isotherm to determine the mean pore radius $r = 3.36\,\mathrm{nm}$.[14] The inset depicts the resulting pore radius r distribution obtained by the BJH model. Note that a small shoulder at $2.78\,\mathrm{nm}$ is visible. Applying the BJH model also yields a pore volume of $V_{\mathrm{por}} = 0.009\,\mathrm{cm}^3$. Thus, the sample material exhibits a porosity of $54\,\%$.

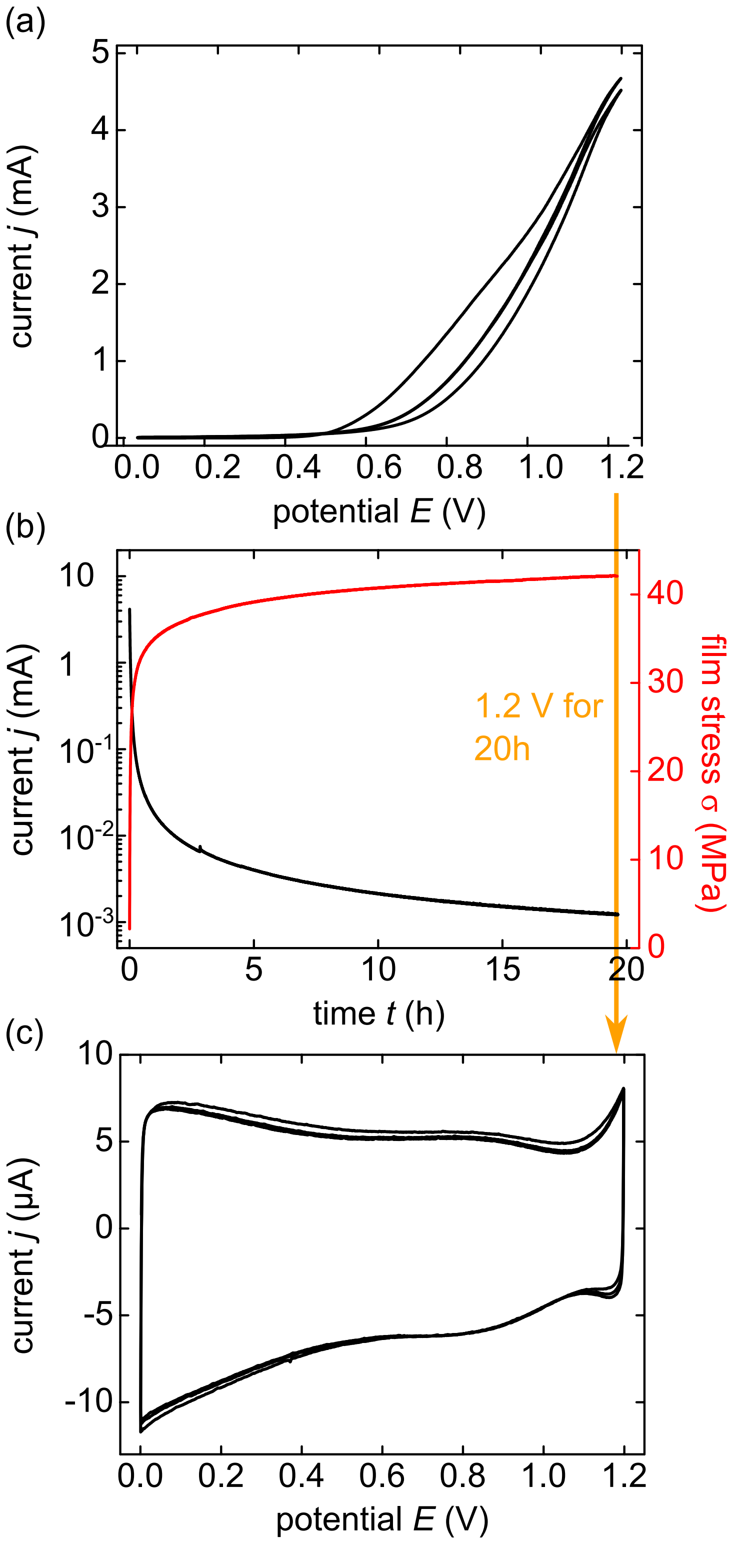

Figure S3: **Initial electrochemical characterisation of porous silicon.** (a) CV measurement with a scan rate of $10\,\mathrm{mVs^{-1}}$ in $1\,\mathrm{mol\,l^{-1}}$ $HClO_4$ electrolyte solution. (b) Constant potential of 1.2 V versus SHE applied to the porous silicon as well as the evolving film stress. (c) CV measurements after the constant potential of 1.2 V with the same parameters as in (a).

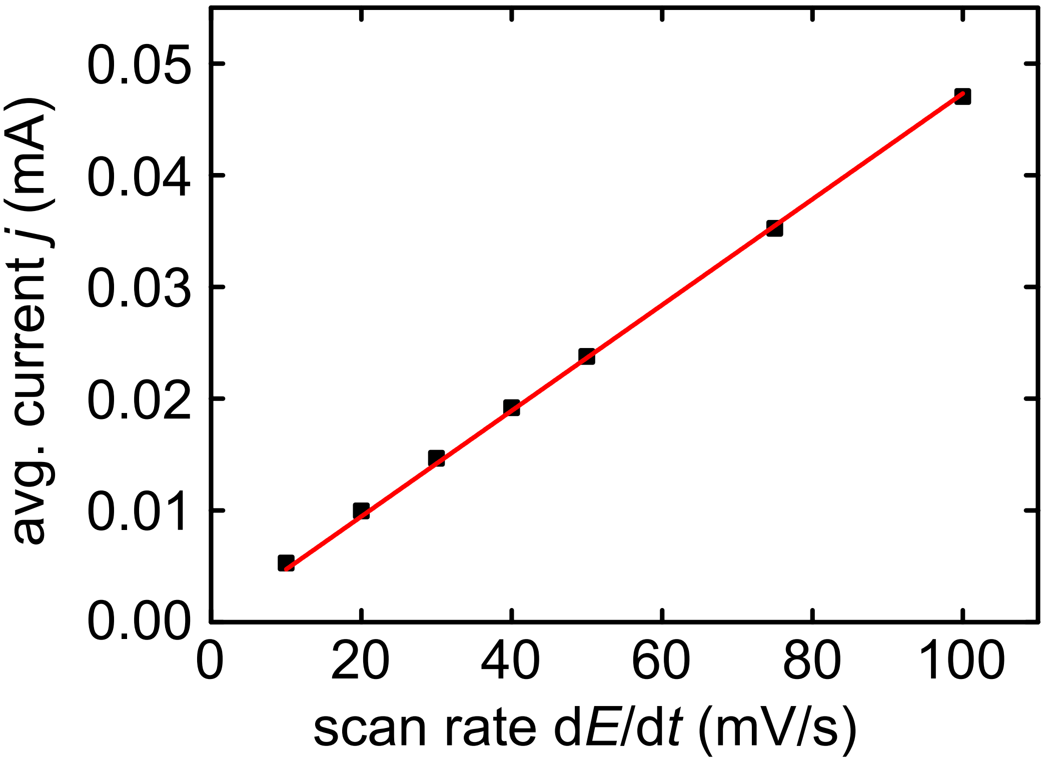

Figure S4: **Performance parameters for the electrochemical in-situ cantilever bending measurement with NaCl electrolyte solution.** The graph depicts the average values for the maximal current of j plotted against varying potential scan rates$\mathrm{d}E/\mathrm{d}t$ from $10\,\mathrm{mV/s}$ to $100\,\mathrm{mV/s}$. The red line indicates a linear regression of the data points which yields the capacitance c as the slope.

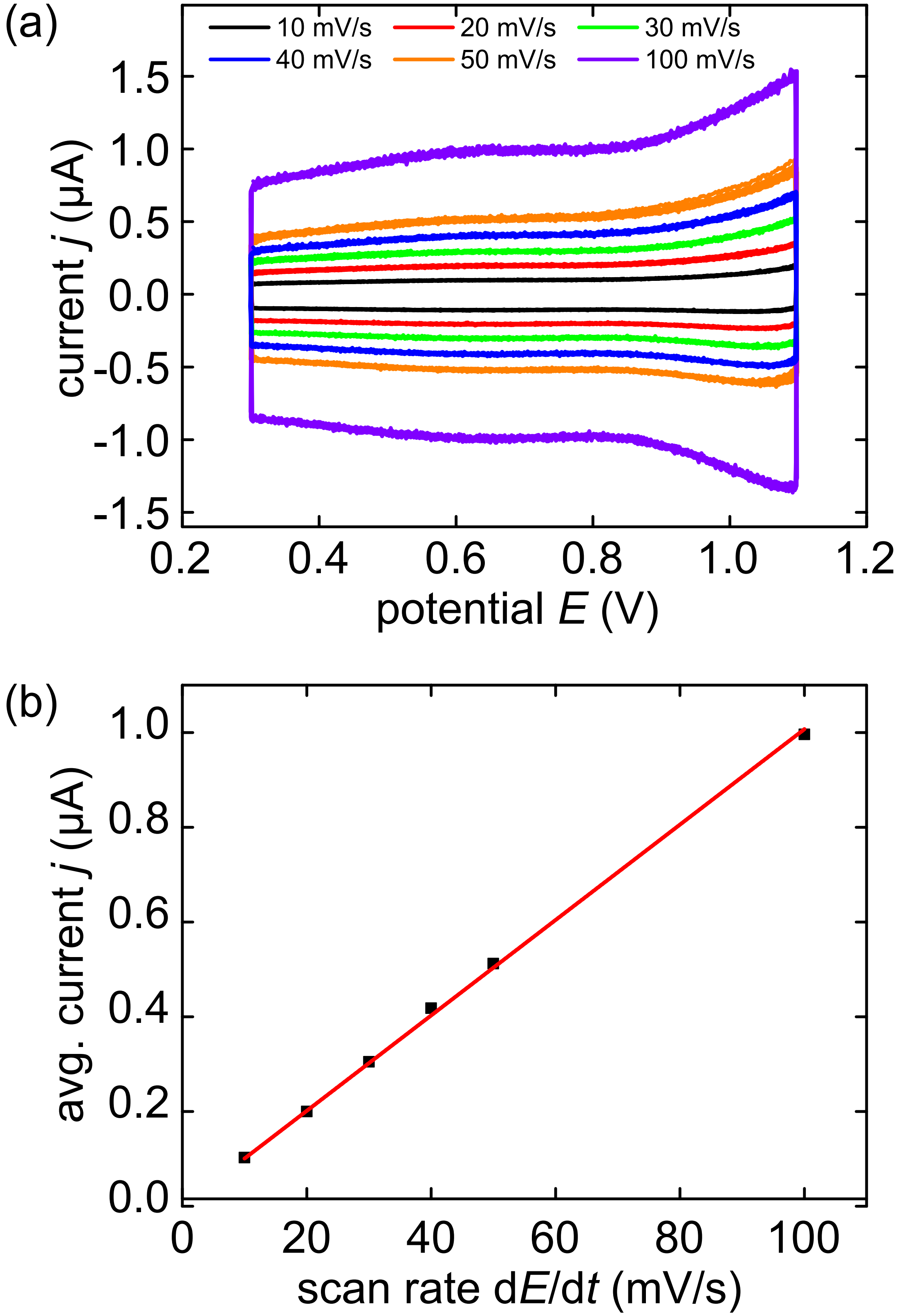

Figure S5: **Electrochemical characterisation of the bulk silicon sample installed in the in-situ cantilever bending setup.** (a) CV measurements with different scan rates in the potential region of 0.3 V to 1.1 V are shown. (b) The graph depicts the mean values for the current of j in the range of 0.6 V to 0.8 V plotted against varying potential scan rates $\mathrm{d}E/\mathrm{d}t$ from $10\,\mathrm{mVs}^{-1}$ to $100\,\mathrm{mVs}^{-1}$. The red line indicates a linear regression of the data points which yields a capacitance of $c = 10.07 \pm 0.08\,\mu\mathrm{F}$.

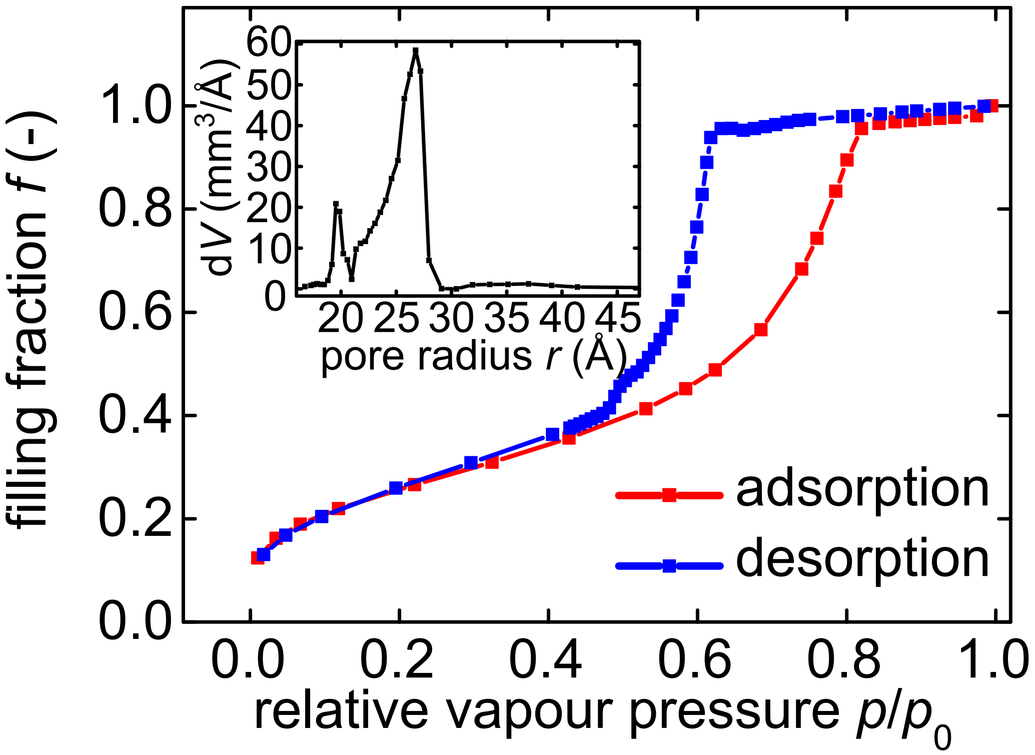

Figure S6: **Nitrogen sorption isotherm of oxidized porous silicon.** Nitrogen sorption isotherm recorded of porous silicon that has been oxidized for 42 hours at 1.2 V. The inset shows the pore radius distribution obtained by an analysis with the BJH model.
